# The Impact of OMEGA-3 Fatty Acids Supplementation on Insulin Resistance and Content of Adipocytokines and Biologically Active Lipids in Adipose Tissue of High-Fat Diet Fed Rats

**DOI:** 10.3390/nu11040835

**Published:** 2019-04-12

**Authors:** Marta Chacińska, Piotr Zabielski, Monika Książek, Przemysław Szałaj, Katarzyna Jarząbek, Iwona Kojta, Adrian Chabowski, Agnieszka Urszula Błachnio-Zabielska

**Affiliations:** 1 Department of Hygiene, Epidemiology and Metabolic Disorders, Medical University of Bialystok, Mickiewicza 2c, 15-089 Bialystok, Poland; paszkiewicziwona89@gmail.com (I.K.); agnieszka.blachnio@umb.edu.pl (A.U.B.-Z.); 2 Department of Physiology, Medical University of Bialystok, Mickiewicza 2c, 15-089 Bialystok, Poland; monika_ksiazek11.89@o2.pl (M.K.); adrian@umb.edu.pl (A.C.); 3 Department of Medical Biology, Medical University of Bialystok, Mickiewicza 2c, 15-089 Bialystok, Poland; piotr.zabielski@umb.edu.pl; 4 Centre for Bioinformatics and Data Analysis, Medical University of Bialystok, Jana Kilińskiego 1, 15-089 Bialystok, Poland; pszemsza@gmail.com; 5 BioStat, Hasselt University, Agoralaan Building D, 3590 Diepenbeek, Belgium; 6 Centre of New Technologies, University of Warsaw, Stefana Banacha 2C, 02-097 Warsaw, Poland; 7 Department of Reproduction and Gynaecological Endocrinology, Medical University of Bialystok, M. Skłodowskiej-Curie 24A, 15-089 Bialystok, Poland; kasia.jarzabek@gmail.com

**Keywords:** insulin resistance, adipose tissue, high-fat diet, omega-3 fatty acids supplementation, biologically active lipids, adipocytokines

## Abstract

It has been established that OMEGA-3 polyunsaturated fatty acids (PUFAs) may improve lipid and glucose homeostasis and prevent the “low-grade” state of inflammation in animals. Little is known about the effect of PUFAs on adipocytokines expression and biologically active lipids accumulation under the influence of high-fat diet-induced obesity. The aim of the study was to examine the effect of fish oil supplementation on adipocytokines expression and ceramide (Cer) and diacylglycerols (DAG) content in visceral and subcutaneous adipose tissue of high-fat fed animals. The experiments were carried out on Wistar rats divided into three groups: standard diet–control (SD), high-fat diet (HFD), and high-fat diet + fish oil (HFD+FO). The fasting plasma glucose and insulin concentrations were examined. Expression of carnitine palmitoyltransferase 1 (CPT1) protein was determined using the Western blot method. Plasma adipocytokines concentration was measured using ELISA kits and mRNA expression was determined by qRT-PCR reaction. Cer, DAG, and acyl-carnitine (A-CAR) content was analyzed by UHPLC/MS/MS. The fish oil supplementation significantly decreased plasma insulin concentration and Homeostatic Model Assesment for Insulin Resistance (HOMA-IR) index and reduced content of adipose tissue biologically active lipids in comparison with HFD-fed subjects. The expression of CPT1 protein in HFD+FO in both adipose tissues was elevated, whereas the content of A-CAR was lower in both HFD groups. There was an increase of adiponectin concentration and expression in HFD+FO as compared to HFD group. OMEGA-3 fatty acids supplementation improved insulin sensitivity and decreased content of Cer and DAG in both fat depots. Our results also demonstrate that PUFAs may prevent the development of insulin resistance in response to high-fat feeding and may regulate the expression and secretion of adipocytokines in this animal model.

## 1. Introduction

Obesity, and health complications stemming from it, such as insulin resistance and type 2 diabetes, have become one of the main causes of mortality in developed countries. Among factors predisposing to the development of insulin resistance, environmental factors, such as the lack of physical activity (sedentary lifestyle) and an improper diet, were distinguished. It is well-established that the consumption of a high-fat diet (HFD) enriched in saturated long-chain fatty acids, as well as a Western diet rich in saturated fats and sugar, leads to the induction of insulin resistance [1,2], whereas a diet rich in polyunsaturated fatty acids (PUFA) may exert a positive influence on human health, including cardiovascular system, brain function, insulin resistance, and prevention of inflammation [3,4,5]. It has been shown that the consumption of a Western diet, which is poor in n-3 polyunsaturated fatty acids (OMEGA-3) and rich in n-6 polyunsaturated fatty acids (OMEGA-6), leads to an increase in the n-6:n-3 ratio, to a range from 10:1 to 20:1 [6]. Another study indicated that high dietary n-6:n-3 PUFA ratio is positively associated with excessive adiposity and worse metabolic profile, specifically regarding insulin and HOMA-IR values in the Mexican population [7]. Overweight or obese women with polycystic ovary syndrome, who have increased consumption of only OMEGA-3 fatty acids, showed lower levels of glucose, insulin, HOMA-IR, and triglycerides (TG) as well as higher levels of adiponectin as compared to the placebo group [8]. The decreased level of triacylglycerol (TAG) and non-esterified fatty acids was observed also in men with metabolic syndrome after the n-3 PUFA supplementation [9]. Couet et al. indicate that dietary fish oil decreases body fat mass and stimulates lipid oxidation in healthy adults [10]. Despite the fact that there is evidence that OMEGA-3 is beneficial, there are still some studies that speak about the unfavorable effect of PUFAs [11,12], especially when OMEGA-3 were used as the primary source of PUFAs to treat patients with type 2 diabetes [13]. In rodents studies, accumulating evidence suggests that, in skeletal muscles, the diet enriched with OMEGA-3 PUFAs leads to improved insulin sensitivity in rats [14], and increased expression of genes regulating glucose metabolism and reduced biologically active lipid content in C57BL6 mice [15]. The latest study indicates that fish oil supplementation, which includes eicosapentaenoic acid (EPA) and docosahexaenoic acid (DHA), improves HFD-induced imbalance of lipid homeostasis not only in muscles but also in rat adipose tissues [16]. Although numerous studies have highlighted the beneficial effect of dietary fish oil, especially in animals’ models, the cellular and molecular mechanism, by which these beneficial actions are exerted, remains unclear. A possible explanation for the positive effect of PUFA on insulin sensitivity may be their effect on the improvement of mitochondrial function [17].

Adipose tissue is a main storage of fatty acids (FA) in the form of triacylglycerols. After entering the cell, FA are activated to long-chain acyl coenzyme A (LCACoA), which are substrates in de novo synthesis of other lipids or in β-oxidation process where they are transported across the mitochondrial membrane as acyl-carnitine (A-CAR), through the enzyme carnitine palmitoyltransferase 1 (CPT1) [18,19]. The imbalance between excessive cellular transport of FFAs and the ability to oxidize them in the mitochondria, may result in the accumulation of biologically active lipids like ceramide (Cer) and diacylglycerols (DAG), which have been shown to inhibit the insulin signaling pathway [20,21,22]. These active lipids are probably involved not only in obesity-associated insulin resistance in skeletal muscle but also adipose tissue. Ceramide content was increased in subcutaneous adipose tissue (SAT) of obese men and women, as compared to lean non-diabetic counterparts [22]. Studies performed on obese non-diabetic and obese diabetic subjects showed an increase in the total Cer, DAG, and LCACoA content in subcutaneous and epicardial adipose tissue as compared to the control group (lean and healthy people) [20]. In addition, a positive correlation has been found between the total content of Cer and DAG in subcutaneous tissue and HOMA-IR (homeostasis model assessment of insulin resistance) [20]. However, another report indicated that the total content of Cer was decreased in SAT from obese non-diabetic and obese diabetic humans compared to lean non-diabetic subjects [21]. To date, there are no data about the content of biologically active lipids in visceral adipose tissue (VAT). The results obtained from the subcutaneous and epicardial fat depots are only a signal that these lipids may affect the development of insulin resistance. Furthermore, the molecular changes in adipose tissue that promote obesity-induced insulin resistance are not completely understood.

Over the last years, adipose tissue has ceased to be seen not only as a fatty acid storage, but started to be perceived as an active endocrine tissue [23]. Adipose tissue synthesizes and secretes, into the bloodstream, a number of compounds referred to as adipocytokines, which play an important role in the glucose and lipid metabolism [24,25]. Among these compounds there are leptin, resistin, and adiponectin. All of these adipocytokines affect the insulin sensitivity in peripheral tissues. Leptin is the best known proinflammatory adipokine, which limits the lipid storage, not only by inhibiting food intake, but also by inducing the release of glycerol from adipocytes by stimulating FA oxidation and decreasing the expression of genes involved in FA and TAG synthesis [26]. Plasma leptin concentration grows with increasing body fat mass and decline with the decrease of body weight and during the low-fat diet [24,27]. On the other hand, chronic leptin administration inverts insulin resistance in the high-fat diet-induced insulin resistant Wistar rats [28]. The physiological role of resistin is to maintain glycaemia during hunger. Thus, the pathological effect is associated with the formation of excessive body fat [25]. It has been shown that six-day resistin treatment elevated plasma glucose and insulin levels, as well as HOMA-IR in wild-type mice, confirming that resistin may induce insulin resistance [29]. Adiponectin is an adipokine which improves insulin sensitivity by increasing glucose uptake and fatty acid oxidation in muscles, as well as by inhibiting gluconeogenesis in liver [24]. Adiponectin has anti-inflammatory properties known to inhibit inflammation by blocking NF-κB activation and reducing such cytokines as TNFα, IL-6, and IL-18 [30]. It has been previously shown that the lack of adiponectin causes insulin resistance [31], which is related to increasing the body mass [32]. Moreover, adiponectin can stimulate activity of ceramidase, which results in a beneficial metabolic effect by the decrease of cellular ceramide content mediated through the enhancement of its catabolism and formation of anti-apoptotic metabolite—sphingosine-1-phosphate (S1P) [33]. A recent report indicates a negative relationship between the total content of ceramide in subcutaneous adipose tissue and plasma adiponectin concentration in women [22]. Other studies showed that EPA increases leptin expression in Wistar rat-isolated adipocytes [34] and adiponectin secretion in rodent models of obesity and human obese subjects [35].

Although, there is some evidence that OMEGA-3 fatty acids may improve systemic insulin sensitivity but little is known about the effect of fish oil supplementation on adipocytokines expression and biologically active lipids accumulation under the influence of high-fat diet-induced obesity. Therefore, the aim of the present study was to examine the effect of fish oil supplementation on ceramide and diacylglycerols content in visceral and subcutaneous adipose tissue of high-fat fed Wistar rats. Moreover, we tried to determine whether the expression and secretion of adipocytokines is altered by HFD feeding and whether supplementation with OMEGA-3 fatty acids affect the expression and secretion of adipocytokines.

## 2. Materials and Methods

### 2.1. Animals and Study Design

The investigation was approved by the Ethical Committee for Animal Experiments at the Medical University of Bialystok (approval code 70/2013 ). The experiments were carried out on male Wistar rats (100–120 g of body weight). They were randomly divided into three groups (*n* = 8 in each group): (1) standard diet fed animals—control (SD), (2) high-fat diet (HFD) fed animals, and (3) high-fat diet + fish oil (HFD+FO) fed rats. Animals were acclimatized for one week prior to beginning of the experiment and housed in standard conditions (21 ± 2 °C, 12:12 h light–dark cycle) with free access to standard chow and water. The animals were fed for 8 weeks with appropriate diet. The HFD+FO diet provided 3.4 kJ% from EPA and DHA. The custom-designed diets (Table 1) were formulated by Research Diets INC D10021806-808 (New Brunswick, NJ, USA) and stored at −80 °C. Total non-esterified and esterified fatty acid concentration in the diets were measured according to Carrapiso et al. [36] protocol after suitable modifications. Individual fatty acid methyl esters (FAMEs) were identified and quantified according to the retention times of standards by GLC (Hewlett-Packard 5890 Series II gas chromatograph, CP-Sil 88 capillary column) (Table S1). The rats body weight was determined in every week during the experiment. At the end of the experiment, rats were anesthetized by intraperitoneal injection of sodium pentobarbital in a dose of 80 mg/kg of body weight. Blood taken from the abdominal aorta was collected in heparinized tubes, centrifuged, the plasma separated and flash-frozen in liquid nitrogen. The subcutaneous and visceral adipose tissue were excised (visceral fat from around liver and subcutaneous fat from abdomen region) and immediately frozen (clamped with aluminum tongs precooled in liquid nitrogen) and then stored at –80 °C until analysis.

### 2.2. HOMA-IR Calculation

To estimate the insulin sensitivity, we measured fasting (12-h fast) plasma glucose and insulin concentration for HOMA-IR calculation. Blood samples were taken from a tail vein and glucose concentration was measured in duplicate using glucometer Accu-Chek (Roche, Germany). To determine insulin concentration, 50 μL of blood sample was taken into heparinized Microvette capillary tube (Sarstedt, Numbrecht, Germany), centrifuged, and measured in duplicate using a solid phase two-site enzyme immunoassay (High Range Rat Insulin ELISA, Mercodia AB, Sweden). The equation for calculating HOMA-IR was as follows: HOMA-IR = (fasting plasma glucose × fasting plasma insulin)/2430, where fasting plasma glucose was in mg/dL and fasting plasma insulin in μU/mL [37].

### 2.3. Adipose Tissue Bioactive Lipids

#### 2.3.1. Ceramide

The content of ceramide was measured using a UHPLC/MS/MS approach according to Blachnio-Zabielska et al. [38]. Briefly, adipose tissue samples (20–30 mg) were pulverized under liquid nitrogen and then homogenized in a solution composed of 0.25 M sucrose, 25 mMKCl, 50 mMTrisand, 0.5 mM EDTA, pH 7.4, with 50 μL of the internal standard solution (C17:0-Cer, Avanti Polar Lipids, Alabaster, AL, USA). Immediately afterwards, 1.0 ml of an extraction mixture (isopropanol: water:ethyl acetate, 30:10:60; *v*:*v*:*v*) was added to each sample. The mixture was vortexed and then centrifuged for 10 min at 2844 g in a cooled centrifuge (4 °C) (Sorvall Legend, RT, Thermo Fisher Scientific, Waltham, MA, USA). The supernatant was transferred into a new tube and the pellet was re-extracted. After centrifugation supernatants were combined and evaporated under nitrogen. The dried samples were reconstituted in 100 μL of LC Solvent A (2 mM ammonium formate, 0.15% formic acid in methanol), vortexed, and transferred into a new tube for further analysis. The following ceramides were analyzed and quantified by means of an Agilent 6460 triple quadrupole mass spectrometer using positive ion electrospray ionization (ESI) source with multiple reaction monitoring (MRM): C14:0-Cer, C16:0-Cer, C18:1-Cer, C18:0-Cer, C20:0-Cer, C22:0-Cer, C24:1-Cer, C24:0-Cer. The chromatographic separation was performed with the use of an Agilent 1290 Infinity ultra-high-performance liquid chromatography (UHPLC). The analytical column was a reverse-phase Zorbax SB-C8 column 2.1 × 150 mm, 1.8 μm. Chromatographic separation was conducted in binary gradient with the use of 2 mM ammonium formate, 0.15% formic acid in methanol as solvent A; and 1.5 mM ammonium formate, 0.1% formic acid in water as solvent B, at the flow rate of 0.4 mL/min.

#### 2.3.2. Diacylglycerols

The content of diacylglycerols was measured on the basis of UHPLC/MS/MS approach according to Blachnio-Zabielska et al. [39]. Diacylglycerols were extracted together with ceramide. 50 μL of the internal standard solution (15:0/15:0-DAG, Avanti Polar Lipids, Alabaster, AL, USA) was added into adipose tissue samples. Then, samples were extracted as described above. The following DAG was analyzed and quantified by means of an Agilent 6460 triple quadrupole mass spectrometer: C18:1/18:2, C16:0/18:2, C16:0/16:0, C16:0/18:1, C18:0/20:0, C18:0/18:1, C18:1/18:1, C18:0/18:2 and C16:0/18:0.

#### 2.3.3. Acyl-Carnitine

The content of acyl-carnitine (C16:0-CAR, C18:1-CAR) was measured according to Sun et al. [40] using UHPLC/MS with C17-carnitine (C17:0-CAR) as an internal standard. A-CAR was analyzed by means of an Agilent 6460 triple quadrupole mass spectrometer with the use of ESI source with selective ion monitoring (SIM). The chromatographic separation was performed on the basis of Agilent 1290 Infinity UHPLC in binary gradient using 2 mM ammonium formate, 0.05% heptafluorobutyric acid in water as solvent A; and acetonitrile as solvent B. The flow rate was 0.4 ml/min, and the gradient conditions were as follows: 0 min at 10% B, 0–16 min 10→80% B, and 16–25 min at 80% B. 

### 2.4. Protein and RNA Isolation

Total RNA and protein were isolated from frozen, powered SAT and VAT using NucleoSpin®RNA/Protein isolation kit (Macherey-Nagel, Bethlehem, PA, USA). Total RNA and proteins were separated on NucleoSpin columns according to the manufacturer’s guidelines.

### 2.5. Western Blot

Following target proteins were quantified using primary antibodies: CPT1 B (Cat. number: NBP1-59576; Novus Biologicals, Littleton, CO, USA) and glyceraldehyde 3-phosphate dehydrogenase (GAPDH) (Cat. number: FL-335; Santa Cruz Biotechnology, Dallas, TX, USA). Values were normalized to GAPDH protein expression measured from parallel runs and expressed as fold changes over control group values. All chemicals and equipment used for immunoblotting were purchased from Bio-Rad (Hercules, CA, USA). Representative WB of visceral and subcutaneous adipose tissue CPT1 B protein is given in Figures S1 and S2, respectively.

### 2.6. Real-Time Quantitative Polymerase Chain Reaction (RT-qPCR)

The quality and quantity of total RNA was determined by 1.5% (*w*/*v*) agarose gel electrophoresis and by using NanoDrop 2000 spectrophotometer (Thermo Scientific) (Table S2). Prior to qPCR, 750 ng of total RNA was DNase I treated (Cat. number: 18068015, Invitrogen, Carlsbad, CA, USA) and then reverse transcribed into cDNA using High Capacity cDNA Reverse Transcription Kit with RNase Inhibitor (Applied Biosystems, Thermo Fisher Scientific, Waltham, MA, USA) on MJ Research Thermal Cycler (Model PTC-200, Watertown, MA, USA). RT-qPCR was performed with the use of 7500 Real-Time PCR System (Applied Biosystems, Thermo Fisher Scientific, Waltham, MA, USA) with the SYBR® Green PCR Master Mix (Applied Biosystems, Thermo Fisher Scientific, Waltham, MA, USA). PCR products were obtained by the amplification of cDNA using specific primers as follows (5′-3′ forward, reverse): CTGGCGATTTTCTCTTCATTC and GTCCTGGAACTTTTGGTGTAAT for adiponectin (NM_144744.3); GCTTTGGTCCTATCTGTCCT and GATACCGACTGCGTGTGT for leptin (NM_013076.3); ATCAAGACTTCAGCTCCCTAC and TGACACATTGTATCCTCACGG for resistin (NM_144741.1); TCTCTGCTCCTCCCTGTTCTA and GGCCAAATCCGTTCACACC for GAPDH (NM_017008.4). The qPCR conditions were as follows: 2 min at 50 °C, 10 min at 95 °C, 15 s at 95 °C, and 1 min at 60 °C for a total of 40 amplification cycles. All PCR products were analyzed by melting curve examination and agarose gel electrophoresis to ensure amplification of a single proper PCR product. The qPCR efficiency was 96%. The efficiency was evaluated by standard curves analysis by performing a 10-fold dilution series of cDNA for each gene. All results were normalized to GAPDH used as the reference gene.

### 2.7. Adipocytokines Concentration

Solid phase quantitative sandwich enzyme immunoassay technique (QuantikineELISA, Rat Total Adiponectin/Acrp30, R&D Systems, Minneapolis, MN, USA) was used to measure the plasma adiponectin concentration (in duplicate). Plasma leptin concentration was measured in duplicate using quantitative enzyme immunometric assay (Leptin (rat), EIA Kit, Enzo Life Sciences, Farmingdale, NY, USA). Plasma resistin concentration was measured in duplicate using a quantitative sandwich enzyme immunoassay (Rat Resistin ELISA, BioVendor, Brno, Czech Republic). All assays were performed according to manufacturers’ protocol.

### 2.8. Statistical Analysis

The relative quantification of gene expression was determined using linear mixed models, where the random effect controlled for the correlation between technical replicates from a single individual. We re-did the analysis using 2^ΔΔCt^ method, which yielded almost identical results in total reaction volume of 10 μL in duplicates. The weight changes over time were modelled using linear mixed-effects models to reflect the fact that the weight measurements from an individual are correlated with one another. For the remaining analyses, the data were first analyzed with the use of one-way ANOVA, and in the case that a null hypothesis of no difference between groups was rejected, pairwise t-tests were used to identify groups that significantly differed from each other. Where applicable, Bonferroni method was applied to correct the multiple testing. We used significance coefficient α = 0.05 for all the tests, which allowed the consideration of the variables statistically significant at *p* < 0.05. Tables and figures were created using Microsoft Office 2013. All calculations were performed using R and LibreOffice Calc. Data are presented as mean ± standard deviation (SD).

## 3. Results

### 3.1. General Characteristic

After eight weeks of treatment, final body weight in the HFD+FO-fed group was significantly increased as compared to the control rats. The fasting glucose concentration increased in HFD and HFD+FO groups by over 20% in both cases. Plasma insulin concentration increased by 53% in HFD group and by 24% in HFD+FO, as compared to the control group. However, insulin concentration in HFD+FO group was significantly lower than in HFD group. Despite the fact that the HOMA-IR value increased significantly in both HFD-fed groups (by almost 90% for HFD group and by 50% for HFD+FO group), the HOMA-IR value in the group supplemented with fish oil was significantly lower than in the HFD-fed animals (Table 2).

### 3.2. Ceramide Content in Visceral and Subcutaneous Adipose Tissue

In visceral adipose tissue, the total content of Cer increased in HFD-fed rats as compared to the SD group (Table 3). Moreover, in the HFD group we noticed elevated content of all measured ceramide species except C14:0-Cer, C16:0-Cer, and C24:1-Cer, compared to the control. Fish oil supplementation increased total ceramide content in VAT as compared to the control. Despite the fact that we did not notice a decrease in the total ceramide content in the HFD+FO group as compared to HFD, all individual ceramide species were significantly reduced besides C14:0-Cer, C16:0-Cer, and C24:1-Cer. C24:1-Cer showed an increase in the fish oil supplemented group compared to HFD. The content of C18:0-Cer, C24:1-Cer, and C24:0-Cer in the HFD+FO group was higher in comparison with SD fed animals. In the case of C16:0-Cer, there were no differences in ceramide content between all treated groups. The C14:0-Cer content decreased in both HFD treated groups as compared to control.

In HFD-fed animals, we observed an increased subcutaneous content of total Cer as well as the C18:0-Cer, C20:0-Cer, C22:0-Cer, and C24:0-Cer compared to the control group. Supplementation with OMEGA-3 fatty acids decreased total ceramide content with comparison in both the control and the HFD group. The content of C16:0-Cer and C18:0-Cer in the HFD+FO group were lower compared to the control and HFD group, while C20:0-Cer and C24:0-Cer was decreased exclusively as compared to HFD. SAT C24:1-Cer content in the HFD+FO group displayed changes analogous to content in VAT, and in both cases we observed an increase compared to SD and HFD group. We also noticed an increase of C14:0-Cer in HFD+FO group compared to HFD and C24:0-Cer in HFD+FO group in comparison to control. Moreover, the C14:0-Cer content decreased in both HFD treated groups as compared to control, whereas C18:1-Cer remains unchanged between all experimental groups.

### 3.3. Diacylglycerol Content in Visceral and Subcutaneous Adipose Tissue

Total content of DAG in visceral adipose tissue has not changed under the high-fat feeding. However, we observed a significant increase in 18:2/18:2-DAG, 18:1/18:2-DAG, 18:0/18:1-DAG, and 18:0/18:0-DAG measured species in comparison with control (Table 4). Lowered content of C16-derived DAGs was also noticed in the HFD-fed animals as compared to standard diet. In the HFD+FO group the total content of visceral DAG significantly decreased in comparison to control and HFD group. Fish oil supplementation significantly decreased the individual molecular species of DAG. The content of 16:0/18:2-DAG, 18:1/18:1-DAG, 16:0/16:0-DAG, 16:0/18:0-DAG, and 16:0/18:1-DAG, in the group with fish oil supplementation, was lower in comparison with the control. The 18:1/18:2-DAG was reduced in the HFD+FO group compared to the SD and HFD groups, whereas 18:0/18:2-DAG and 18:2/18:2-DAG content was decreased exclusively in comparison with HFD-fed rats. There were also differences in 18:0/18:1-DAG, 18:0/18:0-DAG, and 18:2/18:2-DAG content between the HFD+FO group and standard diet fed animals. Higher content of the compounds was noticed in the group with fish oil supplementation. In the HFD+FO group elevated content was also observed in the case of 16:0/16:0-DAG as compared to HFD-fed rats.

In subcutaneous fat tissue, the content of total and C18-derived DAGs increased in the HFD group in comparison with standard diet fed animals, whereas 18:1/18:1-DAG remain unchanged. Moreover, in the HFD group we noticed declined content of C16-derived DAGs besides measured 16:/18:2-DAG species. The total content of DAG in the HFD+FO group was significantly reduced in comparison to the control and HFD. Fish oil supplementation decreased the DAG content in C16:0/16:0-DAG and in all C18-derived DAGs as compared to the HFD group. The highest decrease was observed in 18:1/18:1-DAG (by 38%), 18:0/18:0-DAG (by 33%), 18:0/18:1-DAG and 18:0/18:2-DAG (for both by 32%), 18:1/18:2-DAG (by 26%), and 18:2/18:2-DAG (by 17%). There were also differences in C16-DAG species in the HFD+FO group in comparison to control. The content of all C16-derived diacylglycerols as well as 18:1/18:1-DAG were reduced in the HFD group with fish oil supplementation compared to control, whereas the content of 18:2/18:2-DAG, 18:0/18:1-DAG, and 18:0/18:0-DAG were elevated.

### 3.4. Acyl-Carnitine and Mitochondrial Protein Expression

**Figure 1:**
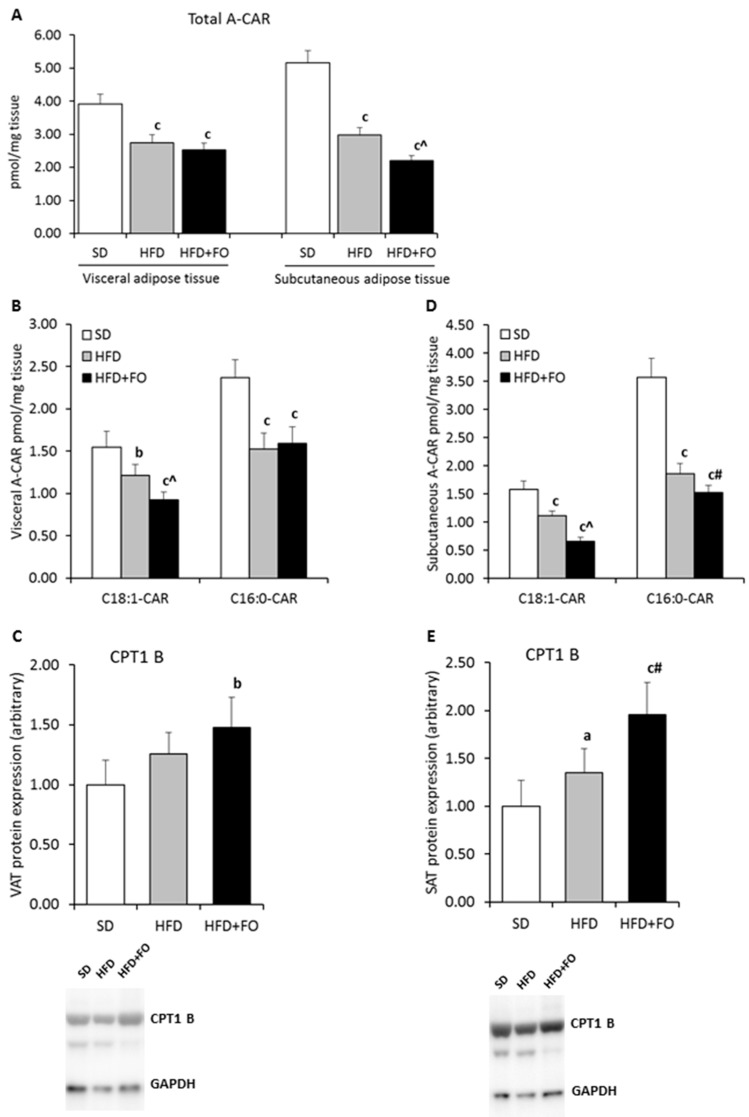
The effect of HFD and HFD+FO consumption on mitochondrial channeling of fatty acids. Panel A presents a total acyl-carnitine (A-CAR) content in rat VAT and SAT, respectively. Panel B and D show the content of A-CAR individual molecular species. Panel C and D present VAT and SAT protein expression of carnitine palmitoyltransferase 1 B (CPT1 B). Values are expressed as mean ± standard deviation. a *p* < 0.05; b *p* < 0.01; c *p* < 0.001—as compared to SD group. # *p* < 0.01; ^ *p* < 0.001—as compared to HFD group, *n* = 8 per group.

Total acyl-carnitine content decreased significantly in visceral and in subcutaneous fat tissue in both HFD groups (Figure 1A–D) as compared to the control. Supplementation of OMEGA-3 fatty acids deepened the decrease of A-CARs in subcutaneous adipose tissue and C18:1-CAR content in visceral adipose tissue, as compared to the HFD group. Despite the observed reduced content of A-CAR, we noticed elevated expression of carnitine palmitoyltransferase 1B (CPT1 B) protein in the HFD and HFD+FO groups in SAT as compared to the control. Fish oil supplementation raised expression of CPT1 B protein in comparison with the HFD and SD groups in SAT and VAT, respectively (Figure 1E,C).

### 3.5. Adipocytokines Expression and Concentration

**Figure 2:**
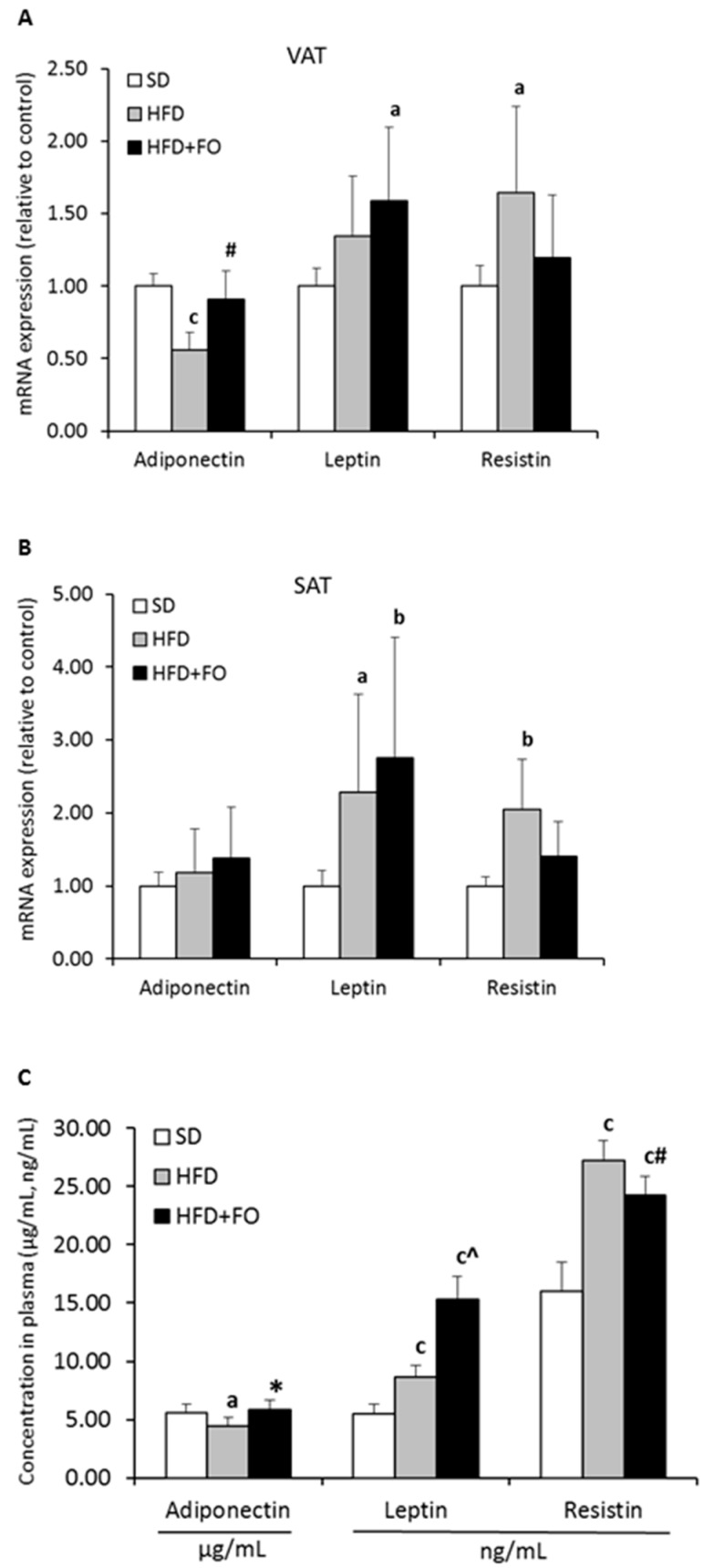
The effect of HFD and HFD+FO consumption on adipocytokines concentration in plasma and mRNA expression in visceral and subcutaneous adipose tissue. Panel A and B present mRNA expression of adipocytokines in VAT and SAT, respectively. Panel C shows, respectively, adiponectin, leptin, and resistin concentration in plasma. Values are expressed as mean ± standard deviation. a *p* < 0.05; b *p* < 0.01; c *p* < 0.001—as compared to SD group. * *p* < 0.05; # *p* < 0.01; ^ *p* < 0.001—as compared to HFD group, *n* = 8 per group.

Adiponectin mRNA expression in VAT was decreased in the HFD group compared to the SD group, whereas in SAT, adiponectin mRNA did not differ among the three groups (Figure 2A,B). Moreover, we noticed significantly lower plasma adiponectin concentration in the HFD group as compared to the control (Figure 2C). In the HFD+FO-fed rats, the visceral mRNA expression of adiponectin and concentration in plasma were increased as compared to the HFD group. The expression of resistin in HFD increased in both VAT and SAT as compared to control animals. Plasma resistin concentration was also higher in the HFD group as compared to the control. In the HFD+FO group, the resistin concentration in plasma was higher than in the control, but lower than in the HFD group. As expected, OMEGA-3 fatty acids supplementation reduces the expression of resistin mRNA in comparison to the HFD-fed group, but this decrease was insignificant. In HFD-fed animals, leptin mRNA was elevated only in subcutaneous adipose tissue as compared to the SD group, whereas fish oil supplementation increases expression of leptin in both tissues. Plasma leptin concentration was higher in both HFD groups, but in the HFD+FO group the level of leptin was even higher than in the HFD group.

## 4. Discussion

Adipose tissue is a metabolically active, integrated unit. Despite the fact that adipose tissue is not the main tissue responsible for insulin-dependent glucose uptake, it appears to be the main tissue responsible for the induction of systemic insulin resistance [20,41,42,43,44]. Recently, it has been demonstrated that OMEGA-3 PUFA, may improve HFD-induced imbalance of lipid homeostasis and prevent the “low-grade” state of inflammation present in adipose tissue of obese individuals [45]. To our knowledge, this is the first report describing the beneficial effect of fish oil supplementation on insulin sensitivity and biologically active lipid content in two main depots of white adipose tissue of high-fat fed Wistar rats. We have found that both types of high-fat diet lead to a significant increase of the HOMA-IR index, fasting blood glucose, and insulin concentration as compared to the standard diet. Previously published data also revealed that male Wistar rats fed HFD developed insulin resistance, which was manifested by elevated fasting blood glucose concentration and increased HOMA-IR index [1,2]. In the present study we have demonstrated that the supplementation of OMEGA-3 fatty acids decreased insulin concentration in plasma and HOMA-IR index in comparison with HFD-treated group, indicating that fish oil improves insulin sensitivity. Similar results were obtained by Kalupahana et al. in male C57BL/6J mice model [46]. They observed significantly higher glycaemia and insulinemia in high-saturated fat (HF) feeding animals compared with the low-fat (LF) diet group, whereas in a group fed HFD with EPA supplementation glycaemia, insulinemia and HOMA-IR were similar to that observed in LF-fed animals [46]. Another animal study showed that the blood glucose concentration was significantly lower in male C57BL6 mice fed HFD with EPA and DHA supplementation, as compared to HFD animals [15]. Emerging evidence suggests that the insulin sensitizing and anti-inflammatory effect is exerted by palmitoleate, which is largely produced in adipose tissue due to increased lipogenesis [47,48]. Moreover, it has been demonstrated that expression of lipogenic enzymes are decreased in adipose tissue of obese insulin-resistant individuals as compared to lean controls. In addition, it has been also shown that bariatric weight loss restored expression of de novo lipogenesis genes in adipose tissue and improved insulin sensitivity [48,49]. Furthermore, lipoprotein lipase (LPL) is an important regulator of FA uptake. It has been found that LPL knockout mice show increase in fatty acid de novo synthesis, especially palmitoleate [50]. In our work we fed animals HFD supplemented with OMEGA-3 PUFA with a significant content of palmitoleate and although the animals did not loss weigh, they had an improved insulin sensitivity comparing to HFD group, which confirms the beneficial role of palmitoleate.

There are just a few studies describing the changes of adipose tissue biologically active lipid content, in obesity-linked insulin resistance [20,22]. In our study we have demonstrated detailed analysis of Cer- and DAG-derived species of lipids. We noticed increased ceramide content in HFD fed animals in both fat depots, whereas content of DAG in HFD group was elevated only in subcutaneous adipose tissue. In both tissues the highest increase was observed in the case of C18-derived measured Cer and DAGs, while the content of C16-derived DAGs dropped in HFD group in comparison to SD. Our findings are consistent with previously obtained results where an increase in total ceramide content in adipocytes in obese females and males than in their lean counterparts has been demonstrated [22]. In contrast to our research, the adipocyte C18:0- and C20:0-Cer content did not differ significantly between obese and lean groups [22], where we noticed almost doubled content in the HFD group in both adipose tissues as compared to the control. In obese men and women, the greater content of adipocytes ceramide species were observed of others containing saturated fatty acids: C14:0-Cer, C16:0-Cer, and C24:0-Cer [22]. However, we also noticed increased content of C24:0-Cer in HFD-fed rats compared to standard diet fed animals in both fat depots. Another study from adipose tissues showed analogous results [20]. The greater total ceramide and diacylglycerol content in obese non-diabetic and obese diabetic subjects was documented, compared to the lean non-diabetic patients in SAT and epicardial adipose tissue [20]. Moreover, elevated content of DAGs was noticed in all particular species in obese individuals in comparison to lean patients, whereas in our study, C16- and C18-derived DAGs were responsible for the decrease and increase of the content of lipids in rats adipose tissues, respectively. In the present research, we focused mainly on the role of the OMEGA-3 PUFA supplementation on Cer and DAG content in adipose tissues of HFD-induced insulin-resistant rats. Our study showed that fish oil supplementation reduced total content of ceramide in subcutaneous adipose tissue and diacylglycerols in both fat depots in comparison with HFD-fed subjects. Interestingly, we noticed significantly decreased content of biologically active lipids in the HFD+FO group versus control animals. It has been previously demonstrated that OMEGA-3 supplementation positively affects the glucose and lipids metabolism and insulin sensitivity [8,14,51]. In the majority of works about fish oil, insulin resistance and obesity, mostly triglycerides, cholesterol, and triacylglycerols, have been taken into consideration. Chiu et al. showed decreased plasma total cholesterol, blood and adipose TG, and low (LDL) and very-low density lipoprotein (VLDL) concentration in HFD-fed rats supplemented with fish oils as compared to HFD group [16]. Normalized plasma triglycerides were noticed in male Wistar sucrose-fed rats where OMEGA-3 PUFA were administered in a dose 0.55g/kg diet [51]. The plasma TG concentration was lower in high fat-fed mice with EPA supplementation group than in the high fat diet alone [46]. Recently published data showed that serum TAG, total cholesterol, LDL, and VLDL, in obese Wistar rats fed diets containing 5% or 20% fish oil, was reduced as compared to obese animals fed lard diets, regardless of the concentration [52]. There are only few reports on the Cer and DAG content in high fat-fed animals after fish oil supplementation [15,53]. Lanza et al. reported that skeletal muscle ceramide content was significantly lower in HFD+FO than observed in HFD. Moreover, HFD+FO diet impaired the HFD-induced increase of muscle C18-Cer content, simultaneously increasing the C14- and C24:1- species, which we also observed in adipose tissue in the present study [15]. Another report indicated that the consumption of α-linolenic acid (ALA), which is the precursor of OMEGA-3 PUFAs, had no effect on epidymal white adipose tissue (EWAT) total TAG, Cer, and DAG content in the supplemented obese Zucker rats, in comparison with lean littermates [53]. In the present study, the consumption of OMEGA-3 fatty acids definitely reduced the total and partial content of particular biologically active lipids in subcutaneous adipose tissue in response to high-fat feeding. The total content of Cer in visceral adipose tissue did not changed in the HFD+FO group as compared to the HFD group; however, the content of individual ceramides changed. In this study, we observed a significant decrease in the content of five ceramides and an increase in only one ceramide—C24:1-Cer, which influenced the increase in the total content of these compounds. In a previously published work, we have shown that the accumulation of ceramides containing 18-carbon fatty acids in their structure has the greatest impact on skeletal muscle insulin sensitivity [1]. It can be assumed that, also in visceral adipose tissue, not all ceramides are responsible for the induction of insulin resistance, but mainly ceramides containing saturated fatty acids. Although the total content of ceramide in the HFD+FO group did not change compared to HFD, adiponectin expression increased and resistin expression decreased in this group, suggesting an improvement of insulin sensitivity, which also strengthens the theory that not all ceramides are responsible for the induction of insulin resistance in visceral fat.

Nevertheless, we hypothesized that OMEGA-3 PUFAs would decrease the accumulation of biologically active lipids, in part by diverting more lipid species on β-oxidation in mitochondria. This hypothesis can be supported by the latest published report, which demonstrated that fish oil supplementation increased gene transcripts for peroxisome proliferator-activated receptor (PPAR) α and γ, as well as genes known to regulate mitochondrial biogenesis in skeletal muscle [15]. To check whether a similar situation occurs in adipose tissue, we measured expression of CPT1 B protein in both fat depots. The expression of CPT1 B protein in HFD+FO group in both adipose tissues was elevated in comparison to SD and also to the HFD group in SAT. Higher expression of CPT1 may result in increased transport of fatty acyl-CoAs to the mitochondria for β-oxidation. Despite the increased expression of CPT1 B protein, in both HFD groups the content of acyl-carnitine decreased in both adipose tissues. Supplementation of OMEGA-3 fatty acids deepens the decrease in both fat depots as compared to HFD group. As mentioned in the introduction, obesity is associated with low-grade inflammation state [54,55]. Adiponectin is associated with sphingolipid metabolism due to receptors that contain ceramidase activity, thereby reducing ceramide levels [33]. In our work we noted reduced plasma adiponectin concentration and mRNA expression in VAT in the HFD group as compared to SD, while the subcutaneous adipose tissue showed no changes in the expression of adiponectin in all groups. Similar results were observed previously [22]. Another study on nondiabetic men and women revealed no significant correlation between serum adiponectin concentration, waist and hip circumferences, and subcutaneous fat area [56]. This strongly suggests that secretion of adiponectin into the bloodstream is not regulated by subcutaneous, but rather by visceral, adipose tissue. On the other hand, we noticed the opposite situation in the HFD group in the case of resistin. The plasma concentrations and mRNA expression of resistin in both adipose tissues were significantly greater in the HFD-fed animals compared to SD-fed littermates. Studies in humans have shown that resistin mRNA expression was elevated in female patients with type 2 diabetes mellitus in comparison to healthy women [57], while the serum concentration of resistin in obese and diabetic patients was significantly increased [58,59]. There are limited data regarding the role of OMEGA-3 on plasma leptin concentration or it’s expression. Most data indicate that OMEGA-3 PUFA lead to a reduction in plasma leptin concentration [27,60]. However, data obtained by Perez-Matute et al. [61] indicated that when rats were fed hyperenergetic HFD with continuous weight gain, the supplementation with OMEGA-3 PUFA resulted in a further increase in leptin levels. Considine et al. also showed that obese individuals have higher plasma leptin levels [62]. In addition, Staiger et al. demonstrated that leptin was positively correlated with waist-to-hip ratio and with both hip and waist circumference. Moreover, in vitro studies indicated that EPA induced increase in leptin mRNA gene expression [63]. In our research we also observed higher plasma leptin concentration as well as increased expression in fat tissues in both HFD groups.

It is well documented that OMEGA-3 supplementation induces an increase in adipocyte adiponectin production [35]. Itoh et al. showed significant increase in plasma adiponectin concentrations in human obese subjects after a three-month treatment with EPA. They also observed that EPA increases adiponectin secretion in genetically obese, leptin deficient mice (ob/ob mice) and HFD-induced obese mice [35]. Another study investigated the plasma adiponectin concentration in male C57BL/6J mice. Plasma adiponectin concentration was comparable in the LF and HF with EPA supplementation groups, suggesting a beneficial role of OMEGA-3 PUFA in insulin resistance induced by HF feeding [46]. Moreover, EPA may have a direct effect on leptin concentration through increasing the production of leptin in rodents and cultured adipocytes [34]. In male C57BL/6J mice fed a high-fat diet with supplementation of Pollock oil, which contains considerable amounts of OMEGA-3 PUFAs, plasma resistin and leptin concentrations were reduced by 14% and 41%, respectively, in comparison to HFD-fed individuals [64]. Our findings mostly confirm those reports. In the HFD+FO group we noticed the elevated plasma adiponectin concentration and expression in VAT as compared to the HFD group, whereas resistin concentration in plasma was significantly reduced in HFD+FO in comparison to HFD animals. Furthermore, leptin concentration and mRNA expression were increased in both adipose tissues in theHFD group with fish oil supplementation.

## 5. Conclusion

HFD-induced insulin resistance is associated with accumulation of biologically active lipid content in adipose tissues. On the other hand, OMEGA-3 fatty acids supplementation improved insulin sensitivity and decreased content of Cer and DAG in both fat rats’ depots. Our results also demonstrate that supplementation with polyunsaturated fatty acids can prevent the development of insulin resistance in response to HFD feeding. This beneficial effect of OMEGA-3 fatty acids supplementation may result, at least in part, from their effect on the expression and secretion of adipocytokines in this experimental animal model.

## Figures and Tables

**Table 1:** Composition of purified experimental diets. Table lists the overall content (g/kg) and the percentage of total energy (kJ%) supplied from proteins, carbohydrates, and fats, and energy density of experimental diets (kJ/g), together with the content (g/kg) and caloricity (kJ) of individual ingredients.

**Component**	**SD**		**HFD**		**HFD+FO**	
	g/kg	kJ%	g/kg	kJ%	g/kg	kJ%
Protein	190	20	260	20	260	20
Carbohydrate	670	70	260	20	260	20
Fat	40	10	350	60	350	60
Energy density, kJ/g	15.9		21.8		21.8	
Individual ingredient	**SD**		**HFD**		**HFD+FO**	
	g/kg	kJ	g/kg	kJ	g/kg	kJ
Casein, 80 Mesh	200	3347	200	3347	200	3347
L-Cystine	3	50	3	50	3	50
Corn Starch	225	3586	0	0	0	0
Maltodextrin 10	125	2092	125	2092	125	2092
Sucrose	68.8	1151	68.8	1151	68.8	1151
Cellulose	50	0	50	0	50	0
Corn Oil	15	565	15	565	15	565
Lard	30	1130	255	9602	193	7268
Menhaden Oil	0	0	0	0	62	2335
Mineral Mix	10	0	10	0	10	0
Dicalcium Phosphate	13	0	13	0	13	0
Calcium Carbonate	5.5	0	5.5	0	5.5	0
Potassium Citrate	16.5	0	16.5	0	16.5	0
Vitamin Mix	10	167	10	167	10	167
Choline Bitartrate	2	0	2	0	2	0

SD—standard diet (control); HFD—high-fat diet; HFD+FO—high-fat diet + fish oil.

**Table 2:** Body weight, fasting plasma glucose and insulin concentration, and HOMA-IR value in Wistar rats fed with different types of diets.

Age	SD	HFD	HFD+FO
	13 weeks	13 weeks	13 weeks
Initial body weight (g)	101.74 ± 15.67	112.14 ± 14.49	119.86 ± 12.33
Final body weight (g)	369.94 ± 27.51	417.12 ± 32.85	435.71 ^b^ ± 60.28
Glucose (mg/dL)	102.50 ± 11.03	127.00 ^c^ ± 4.18	123.50 ^b^ ± 5.43
Insulin (µU/mL)	42.41 ± 3.10	64.73 ^c^ ± 6.88	52.66 ^c#^ ± 2.94
HOMA-IR	1.79 ± 0.24	3.39 ^c^ ± 0.44	2.68 ^c#^ ± 0.22

Values are expressed as mean ± standard deviation. b *p* < 0.01; c *p* < 0.001—as compared to SD group. # *p* < 0.01—as compared to HFD group, *n* = 8 per group. SD—standard diet-control; HFD—high-fat diet; HFD+FO—high-fat diet + fish oil.

**Table 3:** Ceramide content in visceral and subcutaneous adipose tissue in the studied groups (pmol/mg tissue).

***VAT***	**C14:0-Cer**	**C16:0-Cer**	**C18:1-Cer**	**C18:0-Cer**	**C20:0-Cer**	**C22:0-Cer**	**C24:1-Cer**	**C24:0-Cer**	**Total Cer**
SD	0.18 ± 0.02	18.91 ± 2.31	0.08 ± 0.01	1.3 ± 0.15	0.75 ± 0.06	1.79 ± 0.22	2.74 ± 0.36	6.42 ± 0.87	32.17 ± 2.26
HFD	0.13 ^c^ ± 0.01	19.04 ± 2.71	0.12 ^c^ ± 0.01	3.15 ^c^ ± 0.29	1.39 ^c^ ± 0.09	2.82 ^c^ ± 0.28	2.66 ± 0.19	12.05 ^c^ ± 1.19	41.36 ^c^ ± 2.58
HFD+FO	0.14 ^b^ ± 0.01	20.59 ± 1.68	0.07 ^^^ ± 0.00	2.78 ^c*^ ± 0.25	0.78 ^^^ ± 0.08	1.84 ^^^ ± 0.18	4.94 ^c^^ ± 0.40	7.94 ^b^^ ± 0.65	39.08 ^c^ ± 1.50
***SAT***	**C14:0- Cer**	**C16:0- Cer**	**C18:1- Cer**	**C18:0- Cer**	**C20:0- Cer**	**C22:0- Cer**	**C24:1- Cer**	**C24:0- Cer**	**Total Cer**
SD	0.19 ± 0.02	26.35 ± 2.21	0.08 ± 0.01	2.56 ± 0.20	1.05 ± 0.10	1.75 ± 0.13	3.25 ± 0.27	5.41 ± 0.59	40.63 ± 2.41
HFD	0.07 ^c^ ± 0.01	24.79 ± 1.35	0.07 ± 0.01	4.36 ^c^ ± 0.38	1.55 ^c^ ± 0.21	2.11 ^b^ ± 0.21	2.93 ± 0.29	12.26 ^c^ ± 0.30	48.15 ^c^ ± 1.65
HFD+FO	0.12 ^c^^ ± 0.01	19.40 ^c^^ ± 2.14	0.08 ± 0.01	2.25 ^a^^ ± 0.17	1.02 ^^^ ± 0.10	1.88 ± 0.29	4.19 ^c^^ ± 0.33	7.33 ^c^^ ± 0.60	36.28 ^b^^ ± 2.25

Values are expressed as mean pmol/mg tissue ± standard deviation. a *p* < 0.05; b *p* < 0.01; c *p* < 0.001—as compared to SD group. * *p* < 0.05; ^ *p* < 0.001—as compared to HFD group, *n* = 8 per group. SD—standard diet-control; HFD—high-fat diet; HFD+FO—high-fat diet + fish oil; VAT – visceral adipose tissue; SAT – subcutaneous adipose tissue.

**Table 4:** Diacylglycerol content in visceral and subcutaneous adipose tissue in the studied groups (nmol/mg tissue).

***VAT***	**18:2/18:2-DAG**	**16:0/18:2-DAG**	**18:0/18:2-DAG**	**18:1/18:2-DAG**	**16:0/16:0-DAG**	**16:0/18:0-DAG**	**16:0/18:1-DAG**	**18:1/18:1-DAG**	**18:0/18:1-DAG**	**18:0/18:0-DAG**	**Total DAG**
SD	5.61 ± 0.68	3.68 ± 0.49	5.06 ± 1.04	5.81 ± 0.57	4.02 ± 0.51	4.30 ± 0.63	4.69 ± 0.26	6.83 ± 0.88	0.66 ± 0.13	0.15 ± 0.02	40.80 ± 3.27
HFD	10.44 ^c^ ± 0.62	2.92 ^b^ ± 0.25	6.06 ± 0.53	6.68 ^a^ ± 0.61	1.79 ^c^ ± 0.22	2.89 ^c^ ± 0.26	3.02 ^c^ ± 0.26	6.08 ± 0.43	1.35 ^c^ ± 0.13	0.32 ^c^ ± 0.03	41.55 ± 1.91
HFD+FO	7.30 ^c^^ ± 0.68	2.88 ^b^ ± 0.38	4.32 ^^^ ± 0.43	4.66 ^b^^ ± 0.40	2.37 ^c#^ ± 0.30	2.63 ^c^ ± 0.27	3.02 ^c^ ± 0.28	5.28 ^b^ ± 0.75	1.35 ^c^ ± 0.14	0.32 ^c^ ± 0.03	34.14 ^b^^ ± 2.70
***SAT***	**18:2/18:2-D AG**	**16:0/18:2-D AG**	**18:0/18:2-D AG**	**18:1/18:2-D AG**	**16:0/16:0-D AG**	**16:0/18:0-D AG**	**16:0/18:1-D AG**	**18:1/18:1-D AG**	**18:0/18:1-D AG**	**18:0/18:0-D AG**	**Tot al D AG**
SD	6.27 ± 0.55	3.72 ± 0.29	4.91 ± 0.43	4.90 ± 0.36	3.86 ± 0.19	5.06 ± 0.61	4.69 ± 0.15	6.03 ± 0.64	0.87 ± 0.11	0.17 ± 0.02	40.48 ± 0.72
HFD	9.95 ^c^ ± 1.37	3.38 ± 0.52	6.57 ^b^ ± 0.85	6.25 ^a^ ± 0.95	3.12 ^c^ ± 0.26	3.39 ^c^ ± 0.09	3.47 ^c^ ± 0.47	6.86 ± 0.86	2.26 ^c^ ± 0.24	0.39 ^c^ ± 0.06	45.64 ^a^ ± 3.50
HFD+FO	8.19 ^c*^ ± 0.42	2.89 ^b^ ± 0.43	4.42 ^^^ ± 0.61	4.61 ^#^ ± 0.62	2.64 ^c*^ ± 0.34	3.07 ^c^ ± 0.29	3.11 ^c^ ± 0.18	4.23 ^c^^ ± 0.51	1.53 ^c^^ ± 0.15	0.26 ^c#^ ± 0.04	34.95 ^b^^ ± 2.60

Values are expressed as mean nmol/mg tissue ± standard deviation. a *p* < 0.05; b *p* < 0.01; c *p* < 0.001—as compared to SD group. * *p* < 0.05; # *p* < 0.01; ^ *p* < 0.001—as compared to HFD group, *n* = 8 per group. SD—standard diet-control; HFD—high-fat diet; HFD+FO—high-fat diet + fish oil; VAT – visceral adipose tissue; SAT – subcutaneous adipose tissue.

## Supplementary Materials

The following are available online at https://www.mdpi.com/2072-6643/11/4/835/s1, Figure S1: Visceral adipose tissue CPT1 B protein expression with reference gene GAPDH—exposure time 14.0 s; Figure S2: Subcutaneous adipose tissue CPT1 B protein with reference gene GAPDH—exposure time 30.0 s; Table S1: Total fatty acids compositions of purified diets (mg/g)—in situ transesterification; Table S2: The quality and quantity of total RNA in visceral and subcutaneous adipose tissue.
