# Supplementary material for: The Impact of OMEGA-3 Fatty Acids Supplementation on Insulin Resistance and Content of Adipocytokines and Biologically Active Lipids in Adipose Tissue of High-Fat Diet Fed Rats
Source: Nutrients. 2019 Apr 12;11(4):835. doi: 10.3390/nu11040835 (PMC6520951; doi:10.3390/nu11040835)

Table S1. Total fatty acids compositions of purified diets (mg/g)-in situ transesterification.

	**SD**	**HFD**	**HFD+FO**
C14:0 Myristic	2.20 ± 0.20	4.43 ± 0.24	8.51 ± 0.33
C16:0 Palmitic	9.21 ± 1.56	58.25 ± 1.10	56.69 ± 1.55
C16:1 Palmitoleic	0.55 ± 0.12	4.84 ± 0.09	11.98 ± 0.31
C18:0 Stearic	3.55 ± 0.60	27.73 ± 0.43	23.69 ± 0.66
C18:1 Oleic	13.50 ± 2.24	90.83 ± 5.04	75.38 ± 2.06
C18:2 Linoleic	12.42 ± 2.06	50.36 ± 1.04	42.65 ± 1.09
C18:3 Linolenic	0.52 ± 0.09	2.91 ± 0.06	3.06 ± 0.09
C20:0 Arachidic	0.11 ± 0.02	0.41 ± 0.01	0.42 ± 0.02
C20:4 Arachidonic	0.05 ± 0.00	0.43 ± 0.01	0.87 ± 0.02
C20:5 EPA	0.00 ± 0.00	0.03 ± 0.01	8.03 ± 0.19
C22:0 Behenic	0.03 ± 0.00	0.07 ± 0.01	0.15 ± 0.01
C22:6 DHA	0.01 ± 0.01	0.06 ± 0.01	6.46 ± 0.14
C24:0 Lignoceric	0.04 ± 0.00	0.05 ± 0.00	0.09 ± 0.01
C24:1 Selacholeic	0.00 ± 0.00	0.03 ± 0.00	0.19 ± 0.01
**TOTAL**	**42.19 ± 6.85**	**240.43 ± 7.93**	**238.17 ± 6.35**

SD – Standard diet - control; HFD – High-fat diet; HFD+FO – High-fat diet + fish oil;
EPA – Eicosapentaenoic acid; DHA – Docosahexaenoic acid.
Values are expressed as mean ± standard deviation.

Table S2. The quality and quantity of total RNA in visceral and subcutaneous adipose tissue.

**SD**			**HFD**			**HFD+FO**		
Visceral								
	Concentration (μg/μl)	Ratio 260/280		Concentration (μg/μl)	Ratio 260/280		Concentration (μg/μl)	Ratio 260/280
**1.1**	0.1876	2.08	**2.1**	0.1254	2.07	**3.1**	0.0911	2.10
**1.2**	0.1284	2.10	**2.2**	0.1096	2.07	**3.2**	0.0641	2.06
**1.3**	0.1263	2.09	**2.3**	0.0998	2.08	**3.3**	0.0827	2.08
**1.4**	0.1021	2.11	**2.4**	0.0924	2.09	**3.4**	0.0581	2.12
**1.5**	0.0753	2.07	**2.5**	0.0925	2.09	**3.5**	0.0898	2.13
**1.6**	0.1326	2.08	**2.6**	0.1044	2.11	**3.6**	0.0958	2.09
**1.7**	0.0901	2.10	**2.7**	0.1258	1.86	**3.7**	0.0868	2.03
**1.8**	0.0930	2.10	**2.8**	0.1275	2.07	**3.8**	0.0965	2.09
Subcutaneous								
	Concentration (μg/μl)	Ratio 260/280		Concentration (μg/μl)	Ratio 260/280		Concentration (μg/μl)	Ratio 260/280
**1.1**	0.1216	2.10	**2.1**	0.1769	2.09	**3.1**	0.1180	2.07
**1.2**	0.4126	2.06	**2.2**	0.1380	2.08	**3.2**	0.1106	2.10
**1.3**	0.0737	2.12	**2.3**	0.0812	2.12	**3.3**	0.1114	2.08
**1.4**	0.3433	2.07	**2.4**	0.2201	2.10	**3.4**	0.2473	2.10
**1.5**	0.2951	2.07	**2.5**	0.1392	2.09	**3.5**	0.0768	2.07
**1.6**	0.1618	2.10	**2.6**	0.0956	2.11	**3.6**	0.1508	2.10
**1.7**	0.0969	2.09	**2.7**	0.2145	2.10	**3.7**	0.1268	2.10
**1.8**	0.1246	2.10	**2.8**	0.2142	2.09	**3.8**	0.1040	2.09

Western blot analysis

All western blots were performed as described below. "Exposure Time (sec)" was individually set for particular proteins. The sequence of application the samples into the gel wells (n=8 in each group):

1st lane – Protein standard
2nd lane – Empty lane
3rd lane – SD sample
4th lane – HFD sample
5th lane – HFD+FO sample
6th lane… repeats according to the system described above

Figure S1. Visceral adipose tissue CPT1 B protein expression with reference gene GAPDH-exposure time 14.0 s.

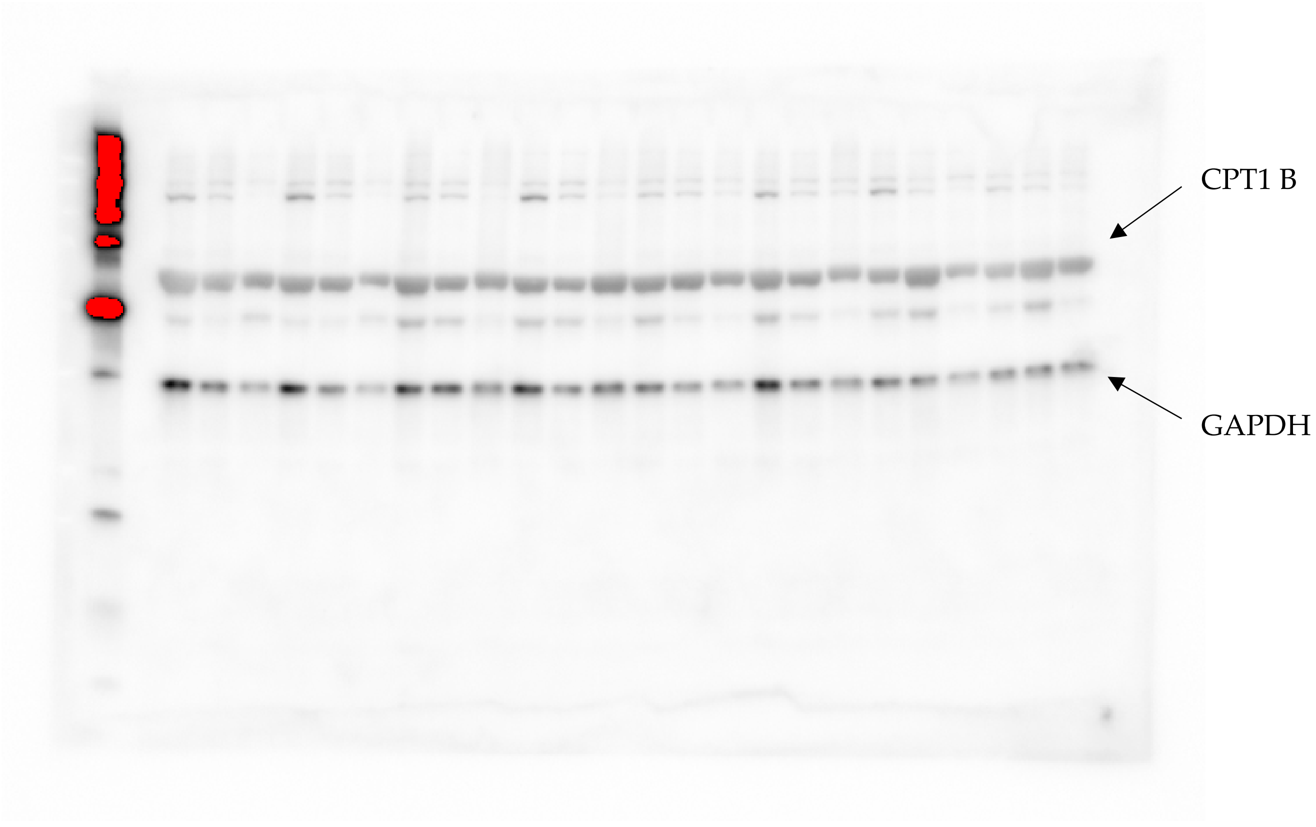

Figure S2. Subcutaneous adipose tissue CPT1 B protein with reference gene GAPDH-exposure time 30.0 s.

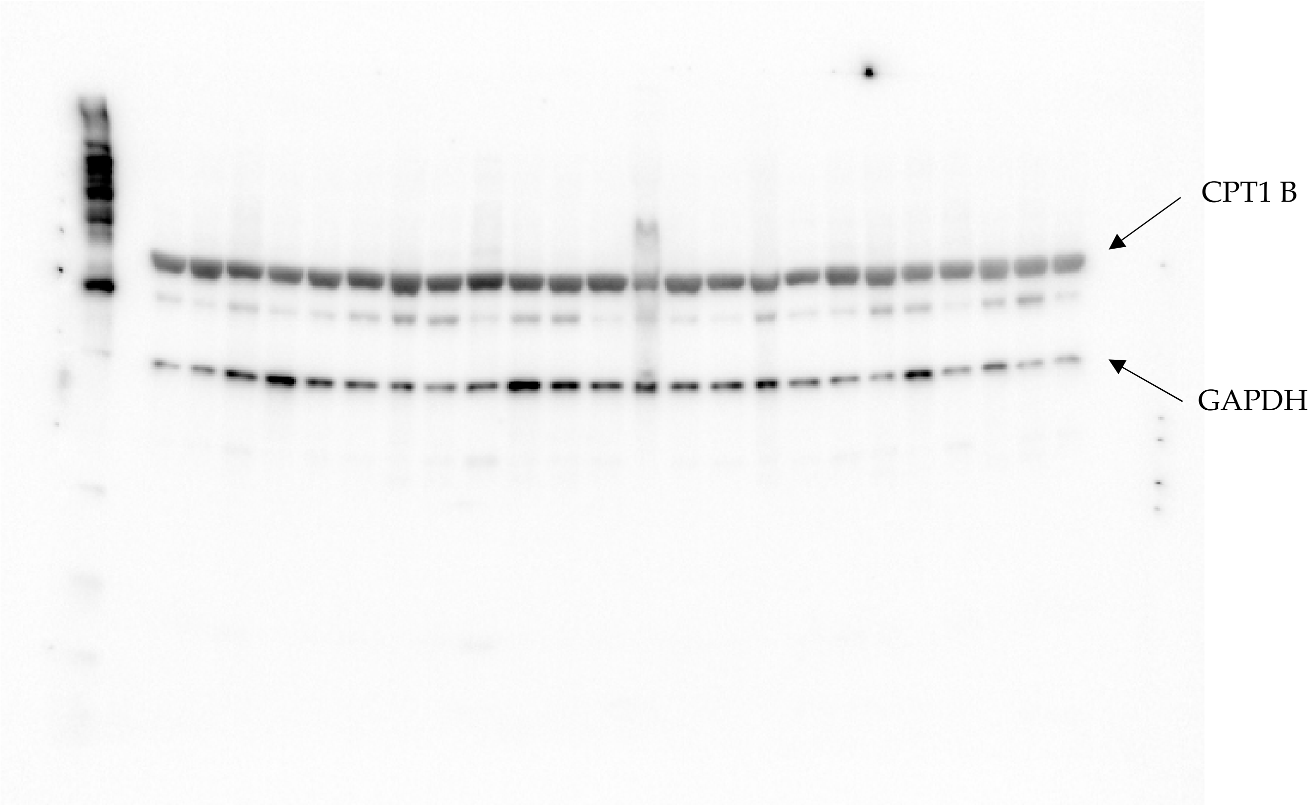

Supplement: Supplementary file 1 [file nutrients-11-00835-s001.pdf]
